# Left Atrial Deformation Parameters After Myocardial Infarction With Low Triiodothyronine Syndrome and Their Prognostic Value

**DOI:** 10.14740/cr2166

**Published:** 2026-02-28

**Authors:** Edita Jankauskiene, Neda Jonaitiene, Martynas Jankauskas, Daiva Emilija Rekiene, Albinas Naudziunas, Giedre Baksyte, Vytautas Zabiela, Diana Zaliaduonyte

**Affiliations:** a Department of Cardiology, Lithuanian University of Health Sciences, Kaunas Clinics, 44307 Kaunas, Lithuania; b Medical Academy, Lithuanian University of Health Sciences, 44307 Kaunas, Lithuania; c The Institute of Cardiology, Lithuanian University of Health Sciences, 44307 Kaunas, Lithuania; d The Kaunas Hospital, Lithuanian University of Health Sciences, 47144 Kaunas, Lithuania

**Keywords:** Speckle-tracking echocardiography, Left atrial deformation, Low T3 syndrome, ST-segment elevation myocardial infarction

## Abstract

**Background:**

Acute myocardial infarction (AMI) management has reduced in-hospital mortality, yet heart failure (HF) and atrial fibrillation (AF) remain common long-term complications. Left atrial (LA) function, assessed via speckle-tracking echocardiography (STE), provides sensitive markers of cardiac remodeling. This study aims to investigate the prognostic value of LA deformation parameters and their significance for long-term outcomes in patients with ST-segment elevation myocardial infarction (STEMI), particularly in relation to low triiodothyronine (T3) syndrome.

**Methods:**

A retrospective study enrolled 140 first-onset STEMI patients treated with primary percutaneous coronary intervention. Thyroid hormone concentrations were measured within 24 h of admission, and patients were classified into low T3 (free triiodothyronine (fT3) < 3.2 pmol/L, n = 44) and control groups (n = 96). Echocardiography and STE were performed within 72 h and repeated after 6 months. LA reservoir strain and conduit and contractile strain rate parameters were analyzed. Long-term outcomes, including AF, rehospitalization, HF, major adverse cardiac events (MACEs), and all-cause death, were assessed after 10 years.

**Results:**

Patients with low T3 syndrome were older, with higher inflammatory markers (P = 0.03) and reduced LA conduit strain rates during the acute phase (P = 0.04). After 6 months, LA volume increased significantly in both groups, but more prominently in low T3 patients (P = 0.03). Reduced LA reservoir strain (area under the curve (AUC), 0.721; P = 0.012) and conduit strain rate (AUC, 0.621; P = 0.012) were strong predictors of MACEs and AF, respectively. Logistic regression identified the LA conduit strain rate, LA reservoir strain, LA volume index, and left ventricular ejection fraction as independent predictors of adverse outcomes.

**Conclusions:**

STE-derived LA deformation parameters provide valuable prognostic information in post-STEMI patients. The LA reservoir strain and LA conduit strain rate are significant predictors of MACEs, while LA global longitudinal strain identifies patients at risk of HF. Early STE evaluation can enhance risk stratification and guide management.

## Introduction

Acute myocardial infarction (AMI) management has significantly reduced the probability of in-hospital mortality [1]. However, heart failure (HF) in survivors of AMI, which requires readmission, remains one of the most common problems. Due to early reperfusion and complementary pharmacological treatment, the development of HF with a reduced ejection fraction (HFrEF) following ST-segment elevation myocardial infarction (STEMI) has substantially decreased. However, the chance of HF with preserved EF (HFpEF) has increased relatively [2]. Therefore, there is a growing need to find new prognostic markers to evaluate cardiac function deterioration after a cardiovascular event.

Ventricular remodeling causes left atrial (LA) enlargement, which is a significant predictor of adverse cardiovascular events [3]. Two-dimensional (2D) speckle-tracking echocardiography (STE) helps evaluate LA longitudinal strain (LALS), i.e., the deformation (stretching or shortening) of the LA myocardium along its long axis. LALS consists of three phases: the reservoir phase (during ventricular systole), conduit phase (during early ventricular diastole), and booster pump phase (during late diastole) [4].

There are several studies that researched the utility and prognostic value of LALS, LA reservoir strain, and LA conduit strain. It has been shown that the mentioned parameters can predict HF, new-onset atrial fibrillation (AF), and major adverse cardiac events (MACEs) after AMI [5].

Cardiovascular disease affects the activity of the hypothalamus–hypophysis–thyroid hormone (TH) cascade and causes a reduction in free triiodothyronine (fT3) concentration within the blood serum. The mechanisms contributing to reduced fT3 levels in AMI may include hypoxia and impaired peripheral conversion of thyroxine (T4) to fT3 mediated by inflammatory processes. Pro-inflammatory cytokines released during AMI can decrease fT3 concentrations by inhibiting type 1 and type 2 deiodinase activity, thereby promoting oxidative stress [6]. These alterations in TH levels are observed in nearly one-third of patients with acute coronary syndrome.

THs may affect LA function, influencing left ventricular (LV) filling pressure, which is linked to increased HF risk [7]. After AMI, low T3 levels—low T3 syndrome—are linked to worse outcomes, including increased mortality, impaired exercise capacity, and adverse cardiac remodeling. Low T3 syndrome is characterized by reduced serum levels of fT3 without increased thyroid-stimulating hormone (TSH) levels during critical illness [8].

Accurate monitoring and management of thyroid function can help prevent adverse outcomes after AMI [7]. Moreover, preliminary studies show that T3 therapy can safely improve cardiac output [9].

The sensitivity of LA function, measured by LA reservoir strain, to diastolic dysfunction (DD) is a common and prognostically significant consequence of myocardial infarction (MI). Post-MI, increased LV stiffness and impaired relaxation elevate LA afterload, reducing LA strain. Advanced imaging techniques like echocardiography and cardiac magnetic resonance (CMR) have shown that LA strain impairment often precedes geometric changes and is a stronger predictor of adverse outcomes, such as AF and HF. Since low T3 syndrome is associated with impaired cardiac performance, it may exacerbate LA strain abnormalities by contributing to DD and delayed cardiac repair mechanisms [10, 11].

## Materials and Methods

### Study design and population

This retrospective clinical study was carried out in the Cardiology Intensive Care Unit within the Cardiology Clinic at the Lithuanian University of Health Sciences Hospital, Kaunas Clinics. One hundred forty patients with first-onset STEMI were included in this study. The patients were recruited between 2011 and 2014. The diagnosis of STEMI was established according to the guidelines of the European Society of Cardiology. The diagnosis of MI with ST-segment elevation was confirmed if the troponin I concentration was higher than the upper normal limit and there was another parameter present: 1) clinical signs of myocardial ischemia; 2) electrocardiographic changes, defined as ST-segment elevation of least two leads ≥ 0.25 mV in men under 40 years, ≥ 0.2 mV in men over 40 years, or ≥ 0.15 mV in women in leads V2–V3, and/or ≥ 0.1 mV in other leads; 3) echocardiographic signs of myocardial ischemia or LV contractility disorder; and 4) coronary artery stenosis or thrombosis confirmed via coronary angiography.

All participants in the study underwent assessment of troponin I and high-sensitivity C-reactive protein (hsCRP) on day 1. The laboratory reference ranges were as follows: troponin I 0–0.04 µg/mL and hsCRP < 7.48 mg/L. We tested troponin I using an automated immunochemical analyzer AIA-2000 LA (TOSOH BIOSCIENCE, Japan). HsCRP was assayed using the Synchron UniCel^®^ DxC 800 automated clinical chemistry system (Beckman Coulter, USA).

Serum TH concentrations were measured on day 1 after the onset of STEMI symptoms, prior to percutaneous coronary intervention (PCI). The following hormones were analyzed: TSH, fT3, and free thyroxine (fT4). Blood samples (5 mL) were collected via venipuncture into vacuum serum tubes with a gel separator. Samples were allowed to clot at 18–25 °C for 15–45 min, after which the serum was separated by centrifugation (1,200 × g for 15 min). TH levels were measured using the automated enzyme immunoassay analyzer AIA-2000 (Tosoh Co., Japan). The laboratory reference intervals were: fT3, 3.2–5.9 pmol/L; fT4, 9–21.07 pmol/L; and TSH, 0.38–4.31 mU/L.

Inclusion criteria were as follows: age 18–80 years; MI with ST-segment elevation within the first 24 h of pain onset and having undergone angioplasty of the “culprit” coronary artery; no history of thyroid pathology; and no medications that affect thyroid function.

Exclusion criteria comprised individuals under 18 years of age and those with a prior history of MI or coronary artery bypass grafting (CABG); thyroid disease; treatment with antithyroid drugs, amiodarone, or glucocorticoids in the last 3 months before hospitalization; current or previous treatment with radioiodine; partial or total thyroidectomy; presence of arrhythmias; moderate to severe valvular heart diseases (including aortic stenosis, aortic regurgitation, known mitral valve disease, or rheumatic heart disorders); and 2D echocardiography images unsuitable for analysis; other severe medical conditions, such as renal failure, chronic liver disease, or cancer; acute infection; and refusal to participate in the study.

The study was conducted in accordance with the ethical standards of the responsible institution on human subjects and with the Declaration of Helsinki and was approved by the Ethics Committee of the Bioethics Centre of Lithuanian University of Health Sciences (Permission No. P3-38/2007).

**Figure 1:**
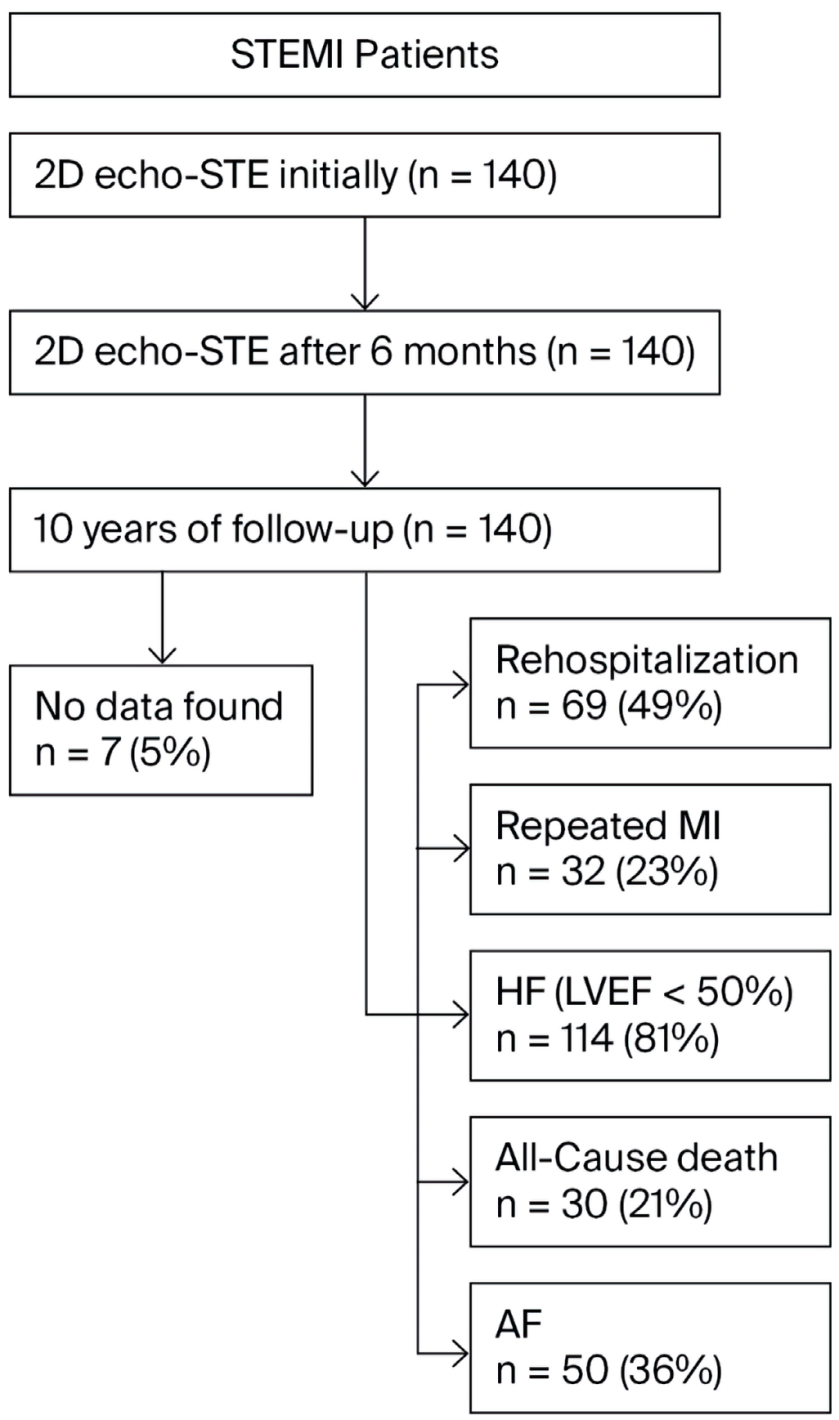
The study design. STEMI: ST-segment elevation myocardial infarction; 2D echo-STE: 2D echocardiography, speckle-tracking echocardiography; MI: myocardial infarction; HF: heart failure; LVEF: left ventricular ejection fraction; AF: atrial fibrillation; 2D: two-dimensional.

A follow-up was performed 10 years after the patient experienced AMI. Patients’ data were evaluated for MACEs: rehospitalization, repeated MI, signs of HF (according to New York Heart Association functional classification and left ventricular ejection fraction (LVEF) < 50%), and all-cause death. We also assessed past or current AF (Fig. 1).

Based on serum fT3 levels at admission, the study population was divided into two groups: the low T3 group (fT3 < 3.2 pmol/L; n = 44) and the control group (fT3 ≥ 3.2 pmol/L; n = 96).

According to the LVEF evaluated within 48–72 h after hospitalization, the study population was divided into three subgroups: group 1 (EF ≥ 50%, n = 26), group 2 (EF ≤ 40%, n = 68), and group 3 (EF 41–49%, n = 46). Group classification was based on the 2021 European Society of Cardiology guidelines for the diagnosis and management of acute and chronic HF, allowing identification of patients with HF with preserved ejection fraction (HFpEF), reduced ejection fraction (HFrEF), and mildly reduced ejection fraction (HFmrEF) [12].

### Echocardiography

All patients with STEMI (n = 140) underwent 2D echocardiography within 48–72 h of hospitalization. Follow-up 2D echocardiography was performed 6 months later to evaluate LV and LA function after AMI (n = 140). Both conventional echocardiography and STE were performed in the left lateral decubitus position using a Vivid 7 ultrasound system (GE VingMed Ultrasound AS; GE Medical Systems, Horten, Norway). Standard images were acquired with a 4.0 MHz transducer in parasternal (long- and short-axis) and apical (two- and four-chamber) views, and measurements were averaged over at least three consecutive cardiac cycles.

Conventional 2D echocardiography was used to assess LV end-systolic and end-diastolic diameters (LVESD, LVEDD), LV end-systolic and end-diastolic volumes (LVESV, LVEDV), LVEF, LA volume, and LA volume index. LVEF was estimated using the biplane Simpson’s method from apical two- and four-chamber views. LA dimensions were measured at end-systole, and LA volume was obtained via Simpson’s method from apical two- and four-chamber views. Morphometric parameters were indexed to body surface area. All echocardiographic parameters were measured in accordance with current guidelines.

Transmitral Doppler inflow was recorded from the apical four-chamber view. Pulsed Doppler measurements included the transmitral early (E) and late (A) diastolic peak flow velocities. Tissue Doppler imaging was used to determine LV myocardial velocities at the mitral annulus during early diastole (E’) in the apical four-chamber view.

### LA strain analysis

Myocardial tissue deformation (strain) was assessed using STE. LA strain and strain rate analyses were performed with EchoPac 6.1 software (GE Medical Systems, Horten, Norway). STE measurements were obtained over three consecutive cardiac cycles, with a mean frame rate of 80–90 frames per second. Prior to speckle-tracking analysis, the timing of aortic and mitral valve opening and closure was determined using pulsed-wave Doppler recordings of aortic and transmitral flows. The interval between two consecutive mitral valve closures was used for regional strain analysis.

Following the guidelines of the European Association of Cardiovascular Imaging and the American Society of Echocardiography task force, strain analysis was conducted in a non-foreshortened apical four-chamber view, with the region of interest (ROI) delineated along the endocardial border [13]. The myocardium was automatically divided into six segments.

LA global reservoir strain, as well as conduit and contractile phase strain rates, were analyzed. These measurements enabled assessment of LA reservoir strain (the change from end-diastole to peak atrial filling), conduit strain rate (the change from peak atrial filling to the onset of atrial contraction), and contractile strain rate (the change from the onset of atrial contraction to end-diastole).

Intra-observer and inter-observer reproducibility of LA strain measurements were assessed using repeated evaluations by the same observer at two separate time points and by a second independent observer. All observers were blinded to prior results and to measurements obtained using the other software package during reproducibility assessment.

Deformation values for each segment were extracted from the results window, and all global deformation indices were calculated as the mean of the measured segmental values.

### Statistical analysis

Statistical analyses were performed using SPSS version 22.0. Quantitative variables are presented as mean ± standard deviation (SD) with 95% confidence intervals (CIs). Sample size calculation was based on a power (β) of 0.8 and a significance level (α) of 0.05.

For normally distributed data, the Student’s *t*-test was used to assess differences in means, whereas the Mann–Whitney test was applied for non-normally distributed data. The Kolmogorov–Smirnov test was used to evaluate normality before applying *t*-tests.

The areas under the receiver operating characteristic (ROC) curves were calculated, and the sensitivity and specificity for potential values of the dependent variable were estimated.

Dependent variables were evaluated using a multivariate binary logistic regression model with a forward selection approach.

A significance level 0.05 was used for hypothesis testing.

## Results

### Patient population

Overall, 44 patients (31.4%) exhibited reduced fT3 levels, while 96 patients (68.6%) had normal fT3 levels. There was no difference in gender between the two study groups. Patients in the low T3 group were older than patients in the control group (P = 0.008). Comorbidities such as stroke, peripheral arterial disease (PAD), and renal insufficiency were observed only in isolated cases between the groups; however, patients in the low T3 group more frequently had dyslipidemia. HsCRP was statistically higher at the low T3 group (P = 0.03). All clinical characteristics of the study are presented in Table 1.

**Table 1:** Clinical Characteristics of the Study Population

Characteristics	Low T3 group (fT3 < 3.2 pmol/L)	Control group (fT3 ≥ 3.2 pmol/L)	P value
Male, n (%)	29 (65.9)	84 (87.5)	0.75
Female, n (%)	15 (34.1)	12 (12.5)	
Age (years)	62.7 ± 9.9	56.3 ± 12.3	0.008
History of hypertension, n (%)	21 (61.8)	58 (60.4)	0.87
Hyperlipidemia, n (%)	21 (61.8)	36 (37.5)	0.014
BMI (kg/m^2^)	27.9 ± 5.5	27.9 ± 4.8	0.96
Troponin I (µg/mL)	41.1 ± 58.2	53.3 ± 84.4	0.46
High sensitivity CRP (mg/L)	23.4 ± 6.9^a^	18.8 ± 3.7^a^	0.03
fT3 (pmol/L)	3.12 ± 0.2	4.05 ± 0.1	0.029
fT4 (pmol/L)	13.75 ± 2.3	14.7 ± 2.8	0.47
TSH (mU/L), median (Q1; Q3)	1.86 (0.82; 3.48)	1.67 (1.23; 8.78)	0.51

^a^Values are presented as mean ± standard deviation. BMI: body mass index; CRP: C-reactive protein; fT3: free triiodothyronine; fT4: free thyroxine; TSH: thyroid-stimulating hormone.

### Echocardiographic measurements

#### Echocardiographic data in acute phase

LVEDV and LVESV were lower in the low T3 group compared to the control group: 101.2 (95.1 ± 107.2) mL vs. 107.9 (104.9 ± 110.7) mL, P = 0.04, and 37.9 (34.0 ± 41.8) mL vs. 43.3 (41.1 ± 45.6) mL, P = 0.006, respectively. In the acute phase of MI, the LA volume and LA volume index showed no statistically significant differences between the groups: 39.3 (34.9 ± 43.7) mL vs. 38.6 (36.6 ± 40.6) mL, P = 0.987, and 20.1 (17.7 ± 22.6) mL/m^2^ vs. 19.2 (18.3 ± 20.1) mL/m^2^, P = 0.845, respectively. Moreover, in the acute phase of MI, the LA conduit phase strain rate was lower in the low T3 group than the control group, respectively—0.8 (0.6 ± 0.9) s^−1^ vs. 0.94 (0.85 ± 1.0) s^−1^, P = 0.04—while other parameters were the same between the groups. The results are shown in Table 2.

**Table 2:** Two-Dimensional, Transvalvular, Tissue Doppler, and Left Atrial Myocardial Deformation Parameters in the Acute Period of Myocardial Infarction

Parameters	Mean values (95% CI)		P value
	Low T3 group (fT3 < 3.2 pmol/L), n = 44	Control group (fT3 ≥ 3.2 pmol/L), n = 96	
LVEDD (mm)	48.9 (46.9 ± 50.8)	48.8 (47.8 ± 49.7)	0.634
LVESD (mm)	39.9 (37.7 ± 42.2)	39.4 (38.5 ± 40.2)	0.549
LVEDV (mL)	101.2 (95.1 ± 107.2)	107.9 (104.9 ± 110.7)	0.040
LVESV (mL)	37.9 (34.0 ± 41.8)	43.3 (41.1 ± 45.6)	0.006
LVEF (%)	40.9 (39.2 ± 42.5)	42.0 (38.5 ± 45.6)	0.364
LA volume (mL)	39.3 (34.9 ± 43.7)	38.6 (36.6 ± 40.6)	0.987
LA volume index (mL/m^2^)	20.1 (17.7 ± 22.6)	19.2 (18.3 ± 20.1)	0.845
E/E’	8.6 (7.1 ± 10.1)	8.4 (7.8 ± 8.9)	0.648
LA reservoir strain (%)	23.1 (18.6 ± 27.7)	22.3 (19.9 ± 24.6)	0.434
LA GLS (%)	22.1 (15.8 ± 28.5)	21.7 (18.4 ± 24.9)	0.641
LA conduit phase strain rate (s^−1^)	0.8 (0.6 ± 0.9)	0.94 (0.85 ± 1.0)	0.040
LA contractile phase strain rate(s^−1^)	0.9 (0.6 ± 1.2)	1.05 (0.9 ± 1.2)	0.425

fT3: free triiodothyronine; T3: triiodothyronine; CI: confidence interval; LVEDD: left ventricular end-diastolic diameter; LVESD: left ventricular end-systolic diameter; LVEDV: left ventricular end-diastolic volume; LVESV: left ventricular end-systolic volume; LVEF: left ventricular ejection fraction; LA: left atrium; E/E’: ratio of early diastolic transmitral flow velocity and early diastolic mitral annular velocity; GLS: global longitudinal strain.

#### Echocardiographic data in 6 months

The LA volume and LA volume index increased after 6 months of AMI in the low T3 group compared to the control group: 56.1 (40.9 ± 71.3) mL vs. 50.9 (47.4 ± 54.5), P = 0.030, and 28.5 (21.6 ± 35.3) mL/m^2^ vs. 25.2 (23.6 ± 26.7) mL/m^2^, P = 0.040, respectively. The results are shown in Table 3. In the low T3 group, the LA volume index increased from 20.1 mL/m^2^ to 28.5 mL/m^2^, a difference of 8.4 mL/m^2^, which is statistically significant, P = 0.007.

**Table 3:** Left Ventricular and Atrial Parameters 6 Months After Myocardial Infarction

Parameters	Mean values (95% CI)		P value
	Low T3 group (fT3 < 3.2), n = 44	Control group (fT3 ≥ 3.2), n = 96	
LVEDD (mm)	51.0 (47.4 ± 54.6)	51.3 (50.1 ± 52.4)	0.588
LVESD (mm)	42.9 (39.4 ± 46.6)	42.3 (41.3 ± 43.4)	0.641
LVESV (mL)	43.6 (34.4 ± 52.7)	47.4 (44.6 ± 50.3)	0.312
LVEDV (mL)	107.3 (94.3 ± 120.3)	111.1 (107.4 ± 114.8)	0.443
EF (%)	40.4 (34.7 ± 46.1)	40.0 (38.2 ± 41.8)	0.451
LA volume (mL)	56.1 (40.9 ± 71.3)	50.9 (47.4 ± 54.5)	0.030
LA index volume (mL/m^2^)	28.5 (21.6 ± 35.3)	25.2 (23.6 ± 26.7)	0.040
E/E’	9.4 (6.9 ± 11.9)	8.6 (7.3 ± 9.9)	0.061

fT3: free triiodothyronine; T3: triiodothyronine; CI: confidence interval; LVEDD: left ventricular end-diastolic diameter; LVESD: left ventricular end-systolic diameter; LVEDV: left ventricular end-diastolic volume; LVESV: left ventricular end-systolic volume; EF: ejection fraction; LA: left atrium; E/E’: ratio of early diastolic transmitral flow velocity and early diastolic mitral annular velocity.

#### Echocardiographic data in groups divided by EF

Moreover, when patients were divided according to EF, LVEDD, LVESD, and LVESV differed significantly between group 1 (EF ≥ 50%) and group 2 (EF ≤ 40%), being higher in patients with lower EF (group 2) during the acute phase of MI and remained the same after 6 months. The LA volume and indexed LA volume differed significantly among the patients, and the differences were within the normal values. The differences are shown in Table 4. Nevertheless, LA reservoir strain differed significantly in the acute phase of MI and was 30.7% (22.1±39.3)% vs. 21.6% (19.8±23.1)% in group 1 and group 2, respectively, P = 0.019. After 6 months of AMI, the LA reservoir strain showed no statistically significant differences between the groups. Nevertheless, the LA conduit phase and LA contractile phase strain rates differed significantly 6 months after MI and were lower in patients with EF ≤ 40% compared to patients with EF ≥ 50%. LA global longitudinal strain (GLS) was significantly lower in patients with EF ≤ 40% after 6 months of AMI (P = 0.048) (Table 5). Moreover, LA GLS decreased significantly after 6 months of AMI. In patients with EF ≥50%, LA GLS in the acute MI phase was 26.9 (19.4 ± 34.5) vs. 25.05 (14.5 ± 35.5), P = 0.216, after 6 months. In patients with EF ≤40%, it was 21.1 (17.4 ± 24.8) and 14.35 (9.3 ± 19.3), respectively, P = 0.038.

**Table 4:** Two-Dimensional, Transvalvular and Tissue Doppler Parameters Between Group 1 and Group 2 in the Acute Period of Myocardial Infarction and After 6 Months of Myocardial Infarction

Parameters		Mean values (95% CI)		P value
		Group 1(EF ≥ 50%), n = 26	Group 2 (EF ≤ 40%), n = 68	
LVEDD (mm)	At baseline	45.7 (44.0 ± 46.5)	50.4 (49.3 ± 51.1)	< 0.0001
	After 6 months	47.7 (46.0 ± 49.5)	52.8 (48.4 ± 53.6)	< 0.0001
LVESD (mm)	At baseline	36.6 (35.1 ± 37.3)	40.7 (39.6 ± 41.3)	< 0.0001
	After 6 months	39.6 (37.7 ± 41.9)	44.3 (43.8 ± 45.2)	0.0003
LVEDV (mL)	At baseline	107.1 (100.5 ± 113.8)	107.4 (104.1 ± 110.8)	0.94
	After 6 months	108.4 (101.2 ± 115.9)	111.6 (106.3 ± 116.9)	0.512
LVESV (mL)	At baseline	33.0 (29.5 ± 34.5)	46.8 (43.9 ± 48.1)	< 0.0001
	After 6 months	37.3 (34.1 ± 40.0)	51.5 (48.8 ± 53.2)	< 0.0001
LVEF (%)	At baseline	54.3 (52.3 ± 55.3)	33.8 (32.7 ± 34.1)	< 0.0001
	After 6 months	51.5 (48.9 ± 54.1)	35.1 (33.9 ± 37.1)	< 0.0001
LA volume (mL)	At baseline	34.3 (30.4 ± 38.2)	40.2 (37.9 ± 42.1)	0.008
	After 6 months	45.1 (37.7 ± 48.3)	53.1 (48.8 ± 57.1)	0.034
LA volume index (mL/m^2^)	At baseline	17.6 (15.9 ± 19.3)	19.8 (18.7 ± 20.9)	0.034
	After 6 months	20.9 (18.6 ± 23.3)	27.0 (24.1 ± 28.8)	0.014
E/E’	At baseline	8.0 (6.7 ± 9.4)	8.5 (7.8 ± 9.3)	0.486
	After 6 months	8.2 (6.2 ± 9.8)	9.3 (7.1 ± 11.5)	0.521

CI: confidence interval; EF: ejection fraction; LVEDD: left ventricular end-diastolic diameter; LVESD: left ventricular end-systolic diameter; LVEDV: left ventricular end-diastolic volume; LVESV: left ventricular end-systolic volume; LVEF: left ventricular ejection fraction; LA: left atrium; E/E’: ratio of early diastolic transmitral flow velocity and early diastolic mitral annular velocity.

**Table 5:** Left Atrial Myocardial Deformation Parameters Between Group 1 and Group 2 in the Acute Period of Myocardial Infarction and After 6 Months of Myocardial Infarction

Parameters		Mean values (95% CI)		P value
		Group 1 (EF ≥ 50%), n = 26	Group 2 (EF ≤ 40%), n = 68	
LA reservoir strain (%)	At baseline	30.7 (22.1 ± 39.3)	21.6 (19.8 ± 23.1)	0.019
	After 6 months	28.1 (19.3 ± 36.9)	23.4 (19.7 ± 27.1)	0.234
LA GLS (%)	At baseline	26.9 (19.4 ± 34.5)	21.1 (17.4 ± 24.8)	0.116
	After 6 months	25.05 (14.5 ± 35.5)	14.35 (9.3 ± 19.3)	0.048
LA conduit phase strain rate (s^−1^)	At baseline	0.8 (0.6 ± 1.0)	0.92 (0.8 ± 1.1)	0.275
	After 6 months	1.14 (0.8 ± 1.4)	0.79 (0.7 ± 0.9)	0.018
LA contractile phase strain rate (s^−1^)	At baseline	1.2 (0.9 ± 1.5)	0.98 (0.8 ± 1.2)	0.104
	After 6 months	1.39 (0.9 ± 1.8)	0.92 (0.7 ± 1.1)	0.036

CI: confidence interval; EF: ejection fraction; LA: left atrial; GLS: global longitudinal strain.

We compared the echocardiographic data between group 1 (EF ≥ 50%) and group 3 (EF 41–49%). The results show that the LVESV and LA indexed volume were higher in group 3 than in group 1 after 6 months post-MI: 42.9 (39.8 ± 45.9) vs. 37.3 (34.1 ± 40.0), P = 0.039, and 25.8 (22.9 ± 28.6) vs. 20.9 (18.6 ± 23.3), P = 0.037, respectively. Nevertheless, LA reservoir strain was lower in group 3 than in group 1 during the acute phase of MI: 22.2 (17.8 ± 26.6) vs. 30.7 (22.1 ± 39.3), P = 0.045. Other strain measurement differences are shown in Table 6.

**Table 6:** Two-Dimensional and Left Atrial Myocardial Deformation Parameters Between Group 1 and Group 3 in the Acute Period and After 6 Months of Myocardial Infarction

Parameters	Mean values (95% CI)		P value
	Group 1 (EF ≥ 50%), n = 26	Group 3 (EF 41–49%), n = 46	
LA reservoir strain (%) in the acute period	30.7 (22.1 ± 39.3)	22.2 (17.8 ± 26.6)	0.045
LVESV (mL) after 6 months	37.3 (34.1 ± 40.0)	42.9 (39.8 ± 45.9)	0.039
LVEF (%) after 6 months	51.5 (48.9 ± 54.1)	42.9 (40.5 ± 45.1)	< 0.0001
LA volume index (mL/m^2^) after 6 months	20.9 (18.6 ± 23.3)	25.8 (22.9 ± 28.6)	0.037
LA conduit phase strain rate (s^−1^) after 6 months	1.14 (0.8 ± 1.4)	0.82 (0.7 ± 0.9)	0.018
LA contractile phase strain rate (s^−1^) after 6 months	1.39 (0.9 ± 1.8)	0.86 (0.6 ± 1.1)	0.022

CI: confidence interval; EF: ejection fraction; LA: left atrial; LVESV: left ventricular end-systolic volume; LVEF: left ventricular ejection fraction.

### Cardiac event predictors

**Figure 2:**
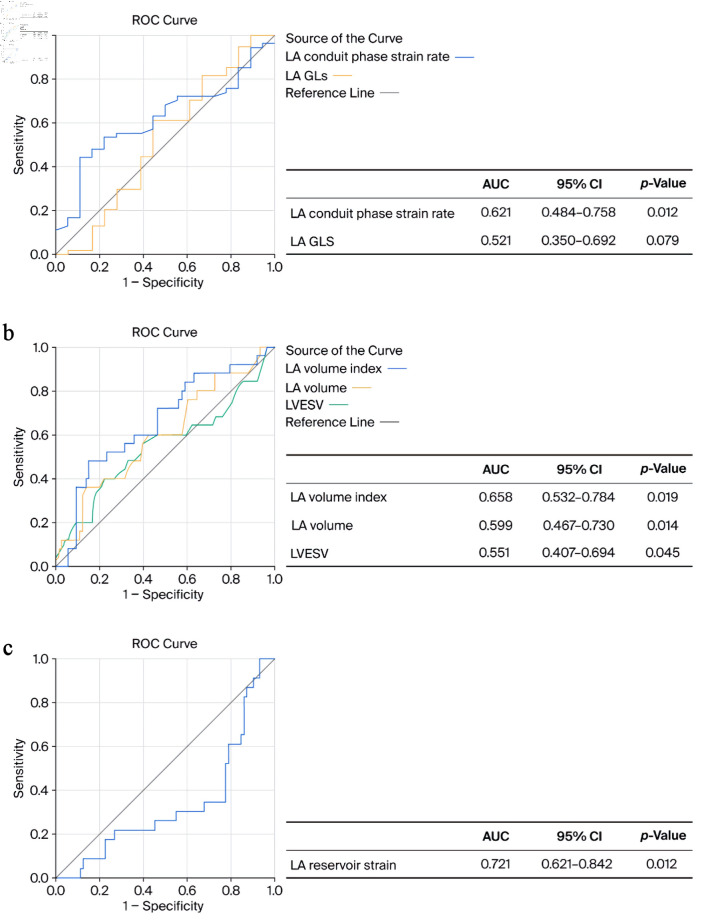
ROC curves for left atrium (LA) conduit phase strain rate and LA global longitudinal strain (GLS) predicting heart failure (a). LA volume index, LA volume, left ventricular end systolic volume (LVESV) (b), and LA reservoir strain (c) predicting all-cause death. AUC: area under the curve; CI: confidence interval; ROC: receiver operating characteristic.

According to the ROC curve, the LA conduit phase strain rate ≤ 0.81 s^−1^, in patients with low T3 syndrome, is statistically significantly associated with LA volume; therefore, it can be stated that the LA conduit phase strain rate is a significant indicator of LA dilatation, LV DD, and future development of AF (sensitivity 83.14) (area under the curve (AUC), 0.63; 95% CI, 0.51–0.75; P = 0.036). After 6 months of MI, LA volume and indexed LA volume significantly increased, although LVEDV did not differ significantly and increased slightly compared to the acute period of MI. It can be argued that the increase in LA parameters indicates an increase in LV filling pressure and could be prognostically significant. Moreover, according to the ROC curve, the LA conduit phase strain rate ≤ 0.95 s^−1^ (sensitivity 84.53) and LA GLS ≤ 16.5% (sensitivity 74.84) were prognostically significant as indicators of HF, P = 0.012 and P = 0.079, respectively (Fig. 2a). According to the ROC curve, LA GLS ≤ 11.8% is prognostically significant in patients with EF ≤ 40% as a prognostic indicator of HF (sensitivity 81.72) (AUC 0.51; 95% CI, 0.34–0.51; P = 0.041). Nevertheless, the ROC curve analysis also showed that the LA volume index ≥ 20.6 (mL/m^2^) (sensitivity 80.24), LA volume ≥ 40.8 (mL) (sensitivity 86.12), and LVESV ≥ 49.5 (mL) (sensitivity 83.68) were statistically significant prognostic predictors of all-cause death, P = 0.019, P = 0.014, and P = 0.045, respectively (Fig. 2b). Also, we found that LA reservoir strain ≤ 19.7% (sensitivity 82.64) is a significant prognostic predictor of all-cause death, P = 0.012 (Fig. 2c).

A multivariable logistic regression analysis was performed to investigate the association between the LA deformation parameter data and the likelihood of MACEs. After adjusting for other echocardiographic data, the results showed that the LA conduit phase strain rate, LA volume index, LA reservoir strain, and LVEF were significantly associated with MACEs. The results are shown in Table 7.

**Table 7:** Multivariate Logistic Regression Analysis Data for Predicting MACEs

Parameters	Exp(B)	95% CI	P
LA conduit phase strain rate	9.971	8.72–11.34	0.008
LA volume index	1.186	0.84–1.26	0.004
LA reservoir strain	0.958	0.72–1.27	0.042
LVEF	0.944	0.81–1.32	0.021

CI: confidence interval; LA: left atrial; LVEF: left ventricular ejection fraction.

According to univariate logistic regression analysis, fT3 < 3.2 pmol/L was a predictive factor for MACEs (specificity 0.660).

## Discussion

Several hypotheses have been proposed to explain the decreased T3 levels that cause dysfunctional LA mechanics during critical illnesses. T3 regulates the expression of sarcoplasmic reticulum Ca^2+^-ATPase (SERCA2a), a pump that transports calcium back into the sarcoplasmic reticulum, and a decrease in T3 level slows the rate of calcium reuptake and impairs relaxation of the atrial muscle [14]. Low T3 promotes structural changes in the atria, a process known as remodeling, and creates a substrate for arrhythmias. TH deficiency promotes the proliferation of cardiac fibroblasts and increases the synthesis of collagen. The resulting collagen deposition in the atrial tissue impairs atrial contraction and relaxation. Low T3 impairs mitochondrial function, reducing cardiac metabolism, which limits the energy supply to the atrial myocardium [15].

In this study, we found that LA remodeling occurred among patients with reduced fT3 levels; the LA volume index increased 8.4 mL/m^2^ (P = 0.007) after 6 months of AMI. Although LA volume and indexed LA volume were numerically higher, they did not show statistically significant differences in the acute period after MI. The main findings were that the LA conduit phase strain rate was lower in the low T3 patient group. It can be proposed that the increase in LA parameters reflects an increase in LV filling pressure. Therefore, it can be considered that the conduit phase LA strain rate can be a prognostic factor of AF after AMI. Similarly, Hoit in 2014 found that an increase in the LA volume index ≥ 8 mL/m^2^ after AMI was an independent predictor of LA remodeling [16]. In 2022, a study performed by Svartstein et al aimed to evaluate the LA strain, conduit phase and reservoir strains in patients with STEMI after PCI and at a 5.6-year follow-up [3]. The LA strain, reservoir and conduit and LA contractile phase strains were all significantly lower in patients with new-onset AF. In our patient population, patients with low T3 syndrome had higher measurements of LVEDV, LVESV, and LA volume and significantly lower LA conduit strain, which can be related to AF development and DD.

Moreover, it is important to evaluate thyroid function in patients with STEMI. Our study revealed that reduced fT3 levels (< 3.2 pmol/L) may be a strong prognostic factor for HF events and all-cause mortality in patients after a first-time STEMI.

A 2018 study demonstrated that patients younger than 75 years with low fT3 levels experienced higher mortality, whereas those with elective or primary PCI and subclinical hypothyroidism had poorer outcomes in terms of repeat revascularization and cardiac death following PCI [2]. Notably, STEMI patients treated with PCI who developed cardiogenic shock exhibited lower fT3 and higher fT4 levels at admission compared to patients without cardiogenic shock. Over a 2.5-year follow-up, individuals with low fT3 (< 2.85 pg/mL) and high fT4 (≥ 0.88 ng/dL) showed the highest all-cause mortality (18.2%), whereas those with high fT3 and low fT4 had the lowest mortality rate (3.8%) [17].

Moreover, we analyzed the value of LA reservoir strain to predict HF events and cardiovascular death in patients after a first-time STEMI. LA reservoir strain was significantly lower in the acute phase of MI in patients with EF ≤ 40%. In our study, LA reservoir strain 19.7% was a prognostic factor for MACEs.

A study performed in 2025 revealed that lower LA reservoir strain was independently associated with an increased risk of HF-related events over a median follow-up period of 8.8 years. Adding the LA strain to clinical risk models significantly enhanced their predictive performance [3]. Furthermore, recent studies indicate that LA strain correlates more closely with LV filling pressure than the LA volume index. A reservoir strain of less than 19–23% is considered abnormally low, and somewhat lower values are used as a marker of elevated LV filling pressure. The relationship between LA strain and filling pressure is most pronounced in patients with reduced LVEF [18]. In 2020, a multicenter magnetic resonance imaging (MRI) study was performed, which found that reduced LA reservoir strain (≤ 22%) and conduit strain (≤ 10%) were independent predictors of MACEs over a median follow-up period of 3.7 years. Incorporating LA strain measurements improved prognostic accuracy beyond traditional markers like LVEF and LA volume [19]. Our study also reported that LA GLS, in particular, decreased after 6 months in patients with EF ≤ 40%, compared to patients with HFpEF. Together, the LA conduit phase strain rate and LA GLS were strong prognostic factors for HF events after STEMI. In 2022, a retrospective study was conducted involving 433 patients who experienced first-time STEMI and underwent PCI [9]. Multivariate Cox hazard analyses identified LA reservoir strain < 25.8% and GLS ≥ –11.5% as significant predictors of MACEs. Adding LA reservoir strain to conventional parameters improved risk stratification, as evidenced by a net reclassification improvement (NRI) of 0.24 (95% CI: 0.11–0.40).

In 2024, a study involving 501 patients with AMI and AF found that LA reservoir strain was independently associated with a composite outcome of all-cause mortality and major adverse cardiovascular events. Notably, in patients with AF, LA reservoir strain remained a significant predictor, whereas LA volume and LV GLS did not [6]. Moreover, our study revealed that lower LA reservoir strain (≤ 19.7%) and conduit strain rate (≤ 0.81 s^−1^) were related to LA dilation; therefore, it may be related to the development of cardiovascular death and AF. In a meta-analysis of 2,542 healthy individuals aged 25–68 years, Pathan et al reported that mean LA reservoir strain was 33.4% in subjects without AF and 24.1% in those who developed incident AF [20].

Moreover, in 2020, Kim et al studied 257 post-MI patients. Each participant underwent both echocardiography and cardiac MRI within a 72-h window to evaluate DD [21]. The study also tracked clinical outcomes, including the onset of AF and HF hospitalizations, over an average follow-up period of 4.4 years. This study compared echo-derived LA strain with MRI-derived measurements in a post-MI population, demonstrating strong agreement between the two modalities and highlighting the superior sensitivity of LA strain over geometric indices in detecting early DD.

In 2024, a study that included a relatively large cohort of 1,409 patients showed that incorporating LA reservoir strain to myocardial (right and left ventricle (RV and LV)) strain helps identify patients at increased mortality risk after AMI (LV GLS: hazard ratio (HR) approximately 1.41 (95% CI 1.06–1.89), P = 0.02; RV GLS: HR approximately 1.48 (95% CI 1.03–2.13), P = 0.04; LA reservoir strain: HR approximately 0.61 (95% CI 0.49–0.76), P < 0.001, accordingly) [22]. A 2019 study of 109 patients admitted to the emergency unit with acute coronary syndrome showed that LA strain—particularly LA reservoir strain—has excellent diagnostic performance in identifying patients with grade III DD, reflecting elevated LV filling pressures [23]. Sonaglioni et al studied patients with acute ischemic stroke in the emergency department who did not have AF. They found that reduced LA reservoir strain, measured by 2D speckle tracking echocardiography, was associated with an increased risk of MACE during a 6-month follow-up. Additionally, lower LA reservoir strain was inversely correlated with the risk of cardioembolic complications, independent of LA size [24]. In 2025, a study of 1,238 patients with STEMI found that impaired LA reservoir strain was 1.5 times more frequent in those who developed new-onset AF and identified an LA reservoir strain < 23% as a crucial factor for LA mechanics [25]. In the study by Beyls et al, LA reservoir strain was an independent predictor of new-onset AF, and an LA reservoir strain level of 27% identified patients at high risk of developing AF after STEMI [26].

Recent studies suggest that STE analysis of LA reservoir strain may be valuable for early risk stratification assessing patients with acute cardiovascular conditions in the intensive care unit.

### Study limitations

This study had several limitations. Firstly, this was a single-center retrospective study with a limited number of patients, especially in the low T3 group. A larger population in the reduced fT3 group could reveal a more detailed prognostic value of LA deformation parameters. LA reservoir strain is a significant prognostic factor for AF in STEMI patients [26]. Additional data are required to evaluate strong predictors of AF in patients with low T3 syndrome after STEMI. Secondly, repeated THs measurements are required during follow-up. In our study THs were measured on the first day before PCI to avoid potential interference from iodinated contrast agents, which may affect fT3 concentrations. Thirdly, larger studies are needed to determine the significance of fT3 as a prognostic marker for MACE, which would be used as a routine test in clinical practice for patients with acute coronary syndrome.

### Conclusions

STE is a technique that can be applied early and is superior to standard echocardiography in assessing LA function among patients with low T3 syndrome and STEMI. In our patient population, it was found that the LA conduit strain rate was lower in the low T3 patient group; however, there is a lack of prognostic evidence regarding the development of AF. LA reservoir strain was significantly lower in the acute phase of MI in patients with EF ≤ 40% and is expected to be a prognostic predictor of mortality (MACE). LA GLS ≤ 11.8% is a prognostically significant indicator of HF in patients with EF ≤ 40%.

## Data Availability

The data presented in this study are available on request from the corresponding author due to privacy and legal restrictions.

## Abbreviations

AMI: acute myocardial infarction
MI: myocardial infarction
HF: heart failure
AF: atrial fibrillation
LA: left atrial
STE: speckle-tracking echocardiography
STEMI: ST-segment elevation myocardial infarction
MACEs: major adverse cardiac events
HFrEF: heart failure with reduced ejection fraction
HFpEF: heart failure with preserved ejection fraction
HFmrEF: heart failure with mildly reduced ejection fraction
LALS: left atrial longitudinal strain
LA GLS: left atrial global longitudinal strain
THs: thyroid hormones
TSH: thyroid-stimulating hormone
fT3: free triiodothyronine
T3: triiodothyronine
fT4: free thyroxine
T4: thyroxine
LV: left ventricular
DD: diastolic dysfunction
CMR: cardiac magnetic resonance
hsCRP: high-sensitivity C-reactive protein
CABG: coronary artery bypass grafting
LVEF: left ventricular ejection fraction
2D echo: two-dimensional echocardiography
LVESD: left ventricular end-systolic diameter
LVEDD: left ventricular end-diastolic diameter
LVESV: left ventricular end-systolic volume
LVEDV: left ventricular end-diastolic volume
E/E’: ratio of early diastolic transmitral flow velocity and early diastolic mitral annular velocity
PAD: peripheral arterial disease
BMI: body mass index
